# Surgical treatment for hepatocellular carcinoma in era of multidisciplinary strategies

**DOI:** 10.1007/s10147-025-02703-7

**Published:** 2025-02-05

**Authors:** Takeshi Takamoto, Yuichirou Mihara, Yujirou Nishioka, Akihiko Ichida, Yoshikuni Kawaguchi, Nobuhisa Akamatsu, Kiyoshi Hasegawa

**Affiliations:** Hepato-Biliary-Pancreatic Surgery Division, Artificial Organ and Transplantation Division, Department of Surgery, Graduate School of Medicine, The University of Tokyo, Tokyo, Japan

**Keywords:** Hepatocellular carcinoma, Liver surgery, Transplantation, Minimally invasive surgery, Immunotherapy

## Abstract

Hepatocellular carcinoma (HCC) remains a significant global health challenge, with over 800,000 new cases diagnosed annually. This comprehensive review examines current surgical approaches and emerging multidisciplinary strategies in HCC treatment. While traditional surgical criteria, such as the Barcelona Clinic Liver Cancer (BCLC) staging system, have been relatively conservative, recent evidence from high-volume Asian centers supports more aggressive surgical approaches in carefully selected patients. The review discusses the evolution of selection criteria, including the new “Borderline Resectable HCC” classification system, which provides more explicit guidance for surgical decision-making. Technical innovations have significantly enhanced surgical precision, including three-dimensional simulation, intraoperative navigation systems, and the advancement of minimally invasive approaches. The review evaluates the ongoing debate between anatomical versus non-anatomical resection and examines the emerging role of robotic surgery. In liver transplantation, expanded criteria beyond the Milan criteria show promising outcomes, while the integration of novel biomarkers and imaging techniques improves patient selection. The role of preoperative and adjuvant therapies is increasingly important, with recent trials demonstrating the potential of immune checkpoint inhibitors combined with anti-VEGF agents in both settings. Despite these advances, postoperative recurrence remains a significant challenge. The review concludes that successful HCC treatment requires a personalized approach, integrating surgical expertise with emerging technologies and systemic therapies while considering individual patient factors and regional variations in practice patterns.

## Introduction

In 2022, almost 860,000 people were diagnosed with liver cancer globally, the most common form of which was hepatocellular carcinoma (HCC) [1]. Liver cancer is the sixth most common cancer and the third leading cause of cancer-related mortality worldwide, after lung and colorectal cancer, showing a relative 5-year survival rate of approximately 20% [2–4]. The similarity between incidence and mortality (830,000 deaths per year) underlines the dismal prognosis associated with this disease. Eastern Asia and sub-Saharan Africa account for about 85% of all cases worldwide, with China alone representing nearly 50% of the global burden (approximately 395,000 new cases annually). In contrast, Northern Europe shows the lowest incidence rates.

Chronic viral hepatitis, particularly hepatitis B virus (HBV) and hepatitis C virus (HCV) infections, remains the predominant risk factor for HCC development globally. The relative contribution of each virus varies by region—HBV infection is highly prevalent in East Asia and Africa, where it is often acquired perinatally or in early childhood. At the same time, HCV predominates in Japan, Europe, and North America [5]. HBV vaccination programs have led to declining HCC incidence in some regions [6]. Similarly, the advent of direct-acting antivirals for HCV has shown promise in reducing HCC risk, though careful surveillance remains important even after viral eradication [5].

With improving control of viral hepatitis in many regions, fatty liver disease has emerged as an increasingly important risk factor for HCC. The rising global prevalence of obesity, diabetes, and metabolic syndrome has led to an epidemic of both non-alcoholic fatty liver disease (NAFLD) [7], and what is now termed metabolic dysfunction-associated fatty liver disease (MAFLD) [8]. While NAFLD has been the traditional terminology based on the exclusion of significant alcohol consumption, MAFLD has recently been proposed as a more inclusive concept that focuses on the presence of metabolic dysfunction regardless of alcohol intake [7, 9].

The MAFLD definition encompasses patients with hepatic steatosis plus one of the following three criteria: overweight/obesity, type 2 diabetes mellitus, or evidence of metabolic dysregulation. This new concept allows for the recognition of both single-etiology MAFLD and mixed-etiology cases where metabolic dysfunction coexists with other liver diseases such as alcohol use or viral hepatitis. Recent data from the Italian Liver Cancer network demonstrated that MAFLD-associated HCC has increased dramatically, now accounting for over 50% of cases in some regions [6]. Unlike viral hepatitis-related HCC, which typically develops in cirrhotic livers, metabolic-associated fatty liver disease-related HCC can occur in the absence of cirrhosis, presenting unique challenges for surveillance strategies [9]. Current evidence suggests that 30–40% of HCC cases in Western countries may be attributable to metabolic liver disease. However, the precise contributions of various risk factors in mixed-etiology cases remain an area of active investigation.

## Update in selection criteria for surgical intervention in HCC

### Tumor-related criteria based on size, number and location

**Fig. 1:**
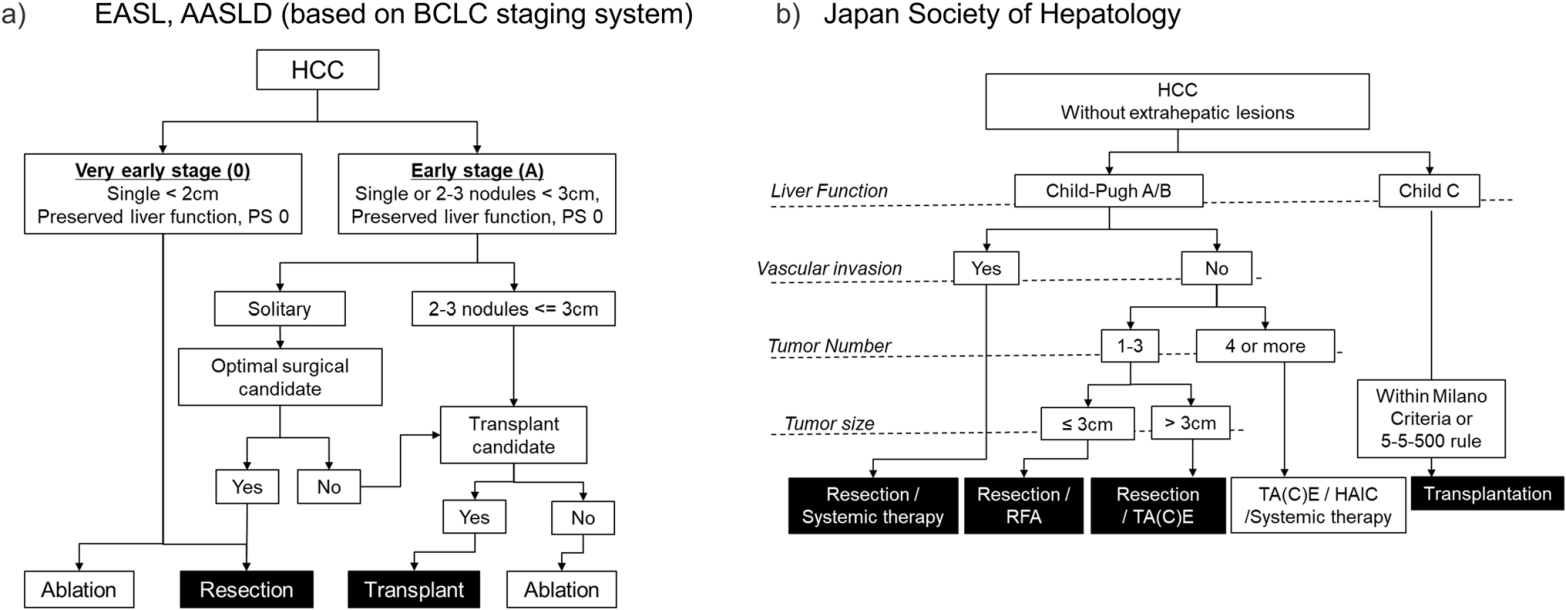
Treatment algorithm for hepatocellular carcinoma (HCC). This figure extracts parts related to surgical treatment. **a** illustrates the overall decision-making process for selecting between resection or transplantation in American Association for the Study of Liver Diseases (AASLD) and European Association for the Study of the Liver (EASL), which based on Barcelona Clinic Liver Cancer (BCLC) staging system. **b** highlights Japanese Society of Hepatoligy (JSH) guidelines, emphasizing that surgical treatment (shown in black with white text) is applicable to a broader range of conditions compared to other criteria. **a** was modified from reference 10 and 17. **b** was modified from reference 16

**Fig. 2:**
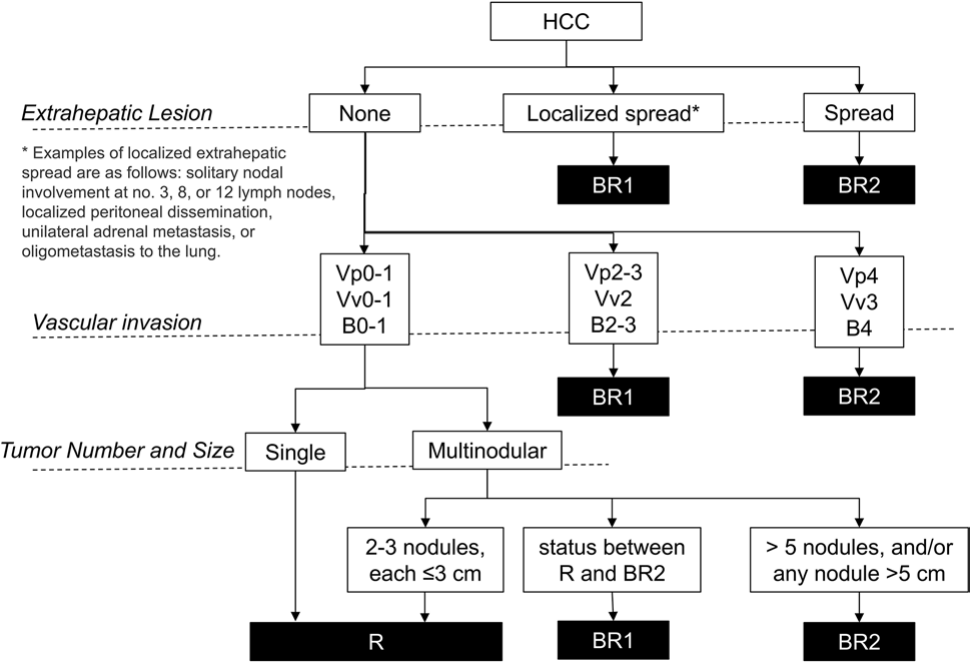
Novel classification criteria for oncologically borderline resectable HCC. The classification system for HCC resectability proposed in reference 22 was reorganized into a flow chart format consistent with conventional criteria

Treatment strategies for hepatocellular carcinoma (HCC) exhibit substantial regional variations, with differing surgical indications across international guidelines [10–18]. The Barcelona Clinic Liver Cancer (BCLC) staging system [10], widely adopted in Western countries, maintains relatively conservative surgical criteria, recommending resection primarily for single tumors without vascular invasion in patients with preserved liver function (Child–Pugh A), absence of clinically significant portal hypertension, and normal bilirubin levels. In contrast, Asian guidelines, particularly those from Japan [16], Hong Kong [19], and Korea [15], allow more aggressive surgical approaches(Table 1). While guidelines used in eastern countries, such as the American Association for the Study of Liver Diseases (AASLD) and the European Association for the Study of the Liver (EASL), follow BCLC criteria, the Japanese Societry of Hepatology has established its own distinct standards that encompass a broader range of resectable cases (Fig. 1). This difference is particularly notable in treatment selection: Western countries often favor liver transplantation, while Japan predominantly pursues hepatic resection, partly due to limited organ availability. Clinical reports and data from high-volume Asian centers have demonstrated favorable long-term outcomes following surgical resection in carefully selected patients with advanced HCC [20, 21], challenging traditional BCLC recommendations. These observations have prompted efforts to better define expanded surgical criteria while maintaining oncological principles. In response to this clinical need and to facilitate evidence-based expansion of surgical indications to better identify such candidates, the Japan Liver Cancer Association and Japanese Society of Hepato-Biliary-Pancreatic Surgery proposed a new classification system including the clear definition of “Borderline Resectable HCC” [22]. This system categorizes patients into three groups: Resectable (R) for single tumors regardless of size or ≤ 3 nodules each ≤ 3 cm; Borderline Resectable 1 (BR1) for intermediate cases; and Borderline Resectable 2 (BR2) for more advanced disease with > 5 nodules or any nodule > 5 cm (Fig. 2). The clinical validation of the BR-HCC definition in the recent proposal [22] has emerged as a topic of significant scholarly discourse.

**Table 1:** Surgical indications for hepatocellular carcinoma across different regions and guidelines worldwide

Region	Country	Year	Guidelines	Liver function factor			Tumor factor	
				Child–Pugh	Portal hypertension	ICG test	Size and number	Vascular invasion
Europe	Europe	2018	EASL [10]	A, B (select cases)	No PH, mild PH in select cases	( +)	Single ≤ 5 cm or ≤ 3 nodules, each ≤ 3 cm	No VI
	Europe	2021	ESMO [14]	A, B (select cases)	No PH	Not specified	Single, any size or ≤ 3 nodules ≤ 3 cm	Not adjacent to vessels or bile duct
USA	USA	2023	AASLD [17]	A, B (select cases)	Minimal/no PH	Not specified	Single ≤ 5 cm or ≤ 3 nodules ≤ 3 cm	No major VI
	USA	2021	NCCN [13]	A, B	Mild/no PH	Not specified	(UNOS criteria)	No major VI
Asia	China	2019	CNHC [19]	A, early B	(−)	ICGR15 < 30%	Resectable, multinodular allowed	Limited VI
	Saudi Arabia	2020	SALT [12]	A, early B	No PH	Not specified	Resectable, multinodular allowed	No VI
	India	2023	INASL [18]	A, B (select cases)	No clinical significant PH	Not specified	B1 and B2 stages for multifocal tumors	No Major VI
	Japan	2022	JSH [16]	A, B	(−)	(+)	Single or ≤ 3 nodules	If resectable
	Korea	2022	KLCA-NCC [15]	A, B (select cases)	Mild/no PH	(+)	Single or ≤ 3 nodules ≤ 3 cm	Limited VI
	Asia–Pacific	2017 2020	APASL [11]	A, B (select cases)	No PH	(+)	Single or multiple small nodules	Limited VI

*ESMO* European Society for Medical Oncology, *AASLD* American Association for the Study of Liver Diseases, *NCCN* National Comprehensive Cancer Network, *CNHC* Chinese National Health Commission, *INASL* Indian National Association for Study of the Liver, *SALT* Saudi Association for the Study of Liver Diseases and Transplantation, *JSH* Japan Society of Hepatology, *KLCA-NCC* Korean Liver Cancer Association—National Cancer Center, *APASL* Asian Pacific Association for the Study of the Liver, *VI* Vascular Invasion

For accurate classification and surgical planning based on these criteria, precise preoperative assessment is crucial. Preoperative radiological imaging is essential for surgical planning in HCC patients. Dynamic enhanced CT provides detailed multiphase liver scans and vascular mapping, crucial for assessing tumor location and vascular invasion. enhanced magnetic resonance imaging (MRI) using Gadolinium ethoxybenzyl-diethylenetriaminepentaacetic acid (Gd-EOB-DTPA) offers superior tissue contrast and detailed tumor characterization through various imaging techniques including diffusion-weighted and contrast-enhanced imaging [23]. While positron emission tomography CT (PET-CT) has lower sensitivity for primary hepatic lesions, it effectively detects extrahepatic metastases [24]. The combined use of these imaging modalities enables precise evaluation of tumor characteristics, vascular involvement, and metastatic disease, allowing surgeons to determine appropriate surgical strategies and optimize treatment outcomes. Furthermore, the appropriateness of excluding unresectable HCC from these criteria remains a subject of rigorous academic debate within the surgical oncology community [25].

### Assessment of liver function and functional reserve

A comprehensive preoperative evaluation encompasses multiple factors beyond tumor characteristics, as total liver resection is not feasible except in cases of liver transplantation. The accurate evaluation of liver functional reserve is essential for safe hepatic resection. The indocyanine green retention test (ICG R15) remains a cornerstone of preoperative assessment, particularly in East Asian countries. Following Makuuchi’s Criteria [26–28] based on ICG R15 values, zero mortality in over 1,000 hepatectomies in the early 2000s has been reported [29]. The criteria suggest ICG R15 values < 10% allow major hepatectomy, while values between 10–20% indicate more limited resection or portal vein embolization are prudent [30, 31]. While ICG R15 provides valuable information about liver functional reserve, it represents only one aspect of millions of hepatic functions. A comprehensive evaluation should include markers of synthetic function such as albumin and cholinesterase. The Albumin-Bilirubin (ALBI) grade has emerged as an objective scoring system that eliminates subjective variables in the Child–Pugh classification and demonstrates good prognostic value [32–34].

Various surgical approaches are available, from limited wedge resection to extended hepatectomy. The choice of procedure must balance oncological radicality with the preservation of adequate future remnant liver. Given the high recurrence rate of HCC and the possibility of repeat resections, parenchymal-sparing hepatectomy, which facilitates future salvage procedures as demonstrated in colorectal cancer liver metastases [35], should be considered when technically feasible [36]. While advanced age itself is not a contraindication for surgery, careful patient selection becomes increasingly important in elderly patients [37]. Recent evidence has identified sarcopenia as an important predictor of post-hepatectomy complications, highlighting the importance of comprehensive functional assessment in the preoperative evaluation [38].

## Surgical techniques and innovations in hepatocellular carcinoma

### Anatomical versus non-anatomical resection

Anatomical resection (AR) following portal vein territory has been advocated mainly in Asia based on the concept that HCC spreads through portal venous branches. AR involves the systematic removal of a hepatic segment containing a tumor along with its portal territory, which is crucial for oncological control of potential microscopic intrahepatic metastases [39].

A systematic review of 14 studies involving 9,444 patients demonstrated that AR was associated with significantly better 5-year overall survival (OR: 1.19; p < 0.001) and recurrence-free survival (OR: 1.26; p < 0.001) compared to non-anatomical resection (NAR) [40]. While AR resulted in longer operating time and greater blood loss, it achieved wider surgical margins and better oncological outcomes, particularly for solitary HCC ≤ 5 cm [28, 41]. However, it should be noted that these studies were retrospective cohort studies, predominantly from Japanese institutions. A well-designed multicenter RCT comparing AR versus NAR is needed to definitively establish the superiority of anatomical resection.

Anatomical resection, including segmentectomy, should be considered the standard approach whenever technically feasible. RFA induces thermal coagulative necrosis in the hepatic parenchyma surrounding the tumor, independent of segmental portal vascular anatomy. This non-anatomical approach provides limited oncological efficacy comparable to non-anatomical partial hepatectomy with respect to the eradication of occult intrahepatic micrometastases. RFA can be considered particularly for small, well-circumscribed HCC that demonstrate a lower probability of portal-mediated intrahepatic metastasis [42].

### Navigation surgery and real-time imaging

Recent technological advances have enhanced surgical precision through preoperative simulation and intraoperative navigation [43–45]. Three-dimensional simulation enables surgeons to understand individual anatomical variations and plan optimal resection planes [43]. This technology has revolutionized preoperative planning by allowing accurate calculation of future liver remnant volume and visualization of the vascular territory. Intraoperative indocyanine green (ICG) fluorescence imaging has emerged as a valuable tool for the real-time identification of tumors and anatomical boundaries [46]. Additionally, real-time virtual sonography that fuses preoperative CT/MRI images with intraoperative ultrasound has enhanced surgical navigation capabilities [47, 48].

### Evolution of minimally invasive surgery and future perspectives

Minimally invasive surgery (MIS) for HCC has evolved significantly. Current evidence demonstrates that laparoscopic liver resection achieves comparable oncological outcomes to open surgery while offering advantages in blood loss, hospital stay, and postoperative recovery [45, 49]. However, laparoscopic approaches are rapidly transitioning to robotic platforms, which offer superior degrees of motion range of the robotic arm and operational simplicity [50, 51]. Robotic systems provide enhanced 3D visualization, precise instrument control, and improved ergonomics, though multiple partial resections (more than 3–5 locations) remain challenging due to time constraints [52]. The emergence of multiple robotic vendors in the market promises increased competition and technological advancement. Recent multi-center studies comparing robotic to laparoscopic and open approaches [53–60] have demonstrated favorable outcomes for robotic surgery, with comparable R0 resection rates, lower conversion rates, and promising long-term survival outcomes (Table 2). Several studies have shown that robotic liver resection achieves comparable oncological outcomes with significantly shorter hospital stays and lower morbidity rates compared to open surgery, even in cases of large HCC.

**Table 2:** Comparison of short- and long-term surgical outcomes for hepatocellular carcinoma by robotic vs laparoscopic, open liver resection

Author	Year	Country	Study design	Diagnosis	Surgery type	Operative time (min)	Blood loss (mL)	R0 resection rate (%)	Conversion rate (%)	Hospital stay (days)	Morbidity (%)	Major complication rate (%)	Long-term Outcome
Lim [53]	2021	Multi-country	Retrospective	HCC	Robotic (44) vs Lap (49)	R: 269, L: 252	Not reported	R: 91%, L: 82%	R: 5%, L: 14%	R: 9, L: 7	R: 16%, L 27%	R 2%, L 4%	3-year OS: R 91%, L 82%; 3-year RFS: R 48%, L 24%
Kato [59]	2022	Japan	Retrospective, single-center	HCC and MET, ICC	Robotic (43) vs Lap (184) vs Open (276)	R: 584, L: 485, O: 495	R: 200, L: 100, O: 350	R: 94.1%, L: 90%, O: 89%	R: 0.8%, L: 5%,	R: 15, L: 12, O: 18	R: 20%, L: 22%, O: 25%	R: 10.8%, L: 12.5%, O: 15%	5-year OS: R 70.3%, L 71.9% 5-year RFS: R 31.9%, O 36.6%
Duong [58]	2022	USA	Retrospective, using NCDB	Stage I HCC	Robotic(123) vs Lap (2,926)	Not reported	Not reported	Not reported	R: 8.1%, L: 3.9%	R: 3, L: 1	Not reported	Not reported	5-year OS: R 63%, L 45%
Chong [57]	2023	Multi-country	PSM	HCC (425) and other tumors	Robotic (107) vs Lap (318), underwent LLS	R: 160, L: 169	R: 50, L: 100	R: 97%, L: 96%	R: 0.6%, L: 5%	R: 5, L: 5	R: 8.4%, L: 11.6%	R: 3.7%, L: 4.2%	Not reported
Zhu [56]	2023	China	PSM	BCLC Stage 0-A HCC	Robotic (71) vs Lap (141) vs Open (157)	R: 220, L: 215, O: 155	R: 200, L: 200, O: 200	R: 98.2%, L: 96.4%, O: 100%	R: 14.3%, L: 12.5%	R: 6, L: 8, O: 12	R: 12.5%, L: 17.9%, O: 23.2%	R: 3.6%, L: 1.8% O: 5.4%	5-year OS: ~ 75%, 5-year RFS: ~ 51%
Lin [54]	2023	China	PSM	Overweight HCC	Robotic (172) vs Open (132)	R: 170, O: 184.5	R: 75, O: 300	R: 99%, O: 98%	R: 2.1%	R: 5, O: 9	R: 4.1%, O: 8.6%	R: 1.9%, O: 1.9%	Not reported
Di Benedetto [58]	2023	Multi-country	PSM	HCC	Robotic (106) vs Open (106)	R: 295, O: 200	R: 200, O: 100	R: 99%, O: 97%	R: 3.2%	R: 4, O: 10	comparable	R: 2.8%, O: 11.3%	2-year OS: R 86.9%, O 83.8%; 2-year RFS comparable
Li [60]	2024	China	PSM	HCC	Robotic (97) vs Lap (244)	R: 210, L: 183.5	R: 100, L: 100	R: 99%, L: 96%	R: 2.1%, L: 7.4%	R: 8, L: 8	R: 4.1%, L: 8.6%	Not reported	5-year OS: R 74.8%, L 80.7%; 5-year RFS: R 58.6%, L 38.3%
Zhang [55]	2024	China	PSM	Large HCC (> 5 cm)	Robotic (309) vs Open (797)	R: 181, O: 201	R: 200, O: 400	R: 98%, O: 97%	R: 0%	R: 6, O: 9	R: 2%, O: 6%	Not reported	5-year OS: R 55.9%, O 53.2%; 5-year RFS: R 26%, O 25.6%

Gray-colored cells indicate statistically significant differences between groups (p < 0.05)

*NCDB* national cancer database in USA, *LLS* left lateral sectionectomy, *OS* overall survival, *RFS* recurrence free survival

### Future directions in surgical innovation

HCC presents distinctive characteristics that fundamentally differentiate its management from other malignancies: the necessity of preserving sufficient functional liver volume in the context of underlying cirrhosis, and its high recurrence rate where repeat resection and other salvage treatments demonstrate therapeutic efficacy. Demographic transitions toward an increasingly aging population portend a substantial rise in cirrhotic patients who exceed conventional age criteria for liver transplantation [61]. For these patients, particularly those who are not transplant candidates due to advanced age, the surgical strategy must focus on minimally invasive, parenchyma-sparing hepatectomy that maintains adequate functional liver volume. The challenge is particularly evident in repeat hepatectomies, where liver regeneration alters the anatomical landscape, necessitating sophisticated navigation systems to visualize optimal resection planes. Furthermore, advances in anti-adhesion biomaterials may facilitate repeat hepatectomies by reducing the technical burden of adhesiolysis [62]. In response to these specific surgical needs in the near future, technological innovation continues to advance rapidly.

The integration of artificial intelligence (AI) with surgical technology continues to advance. AI-assisted surgical planning, augmented reality navigation, and computer vision systems enhance surgical precision. While current challenges include high costs and the need for specialized training, the expanding robotic marketplace and ongoing technological developments suggest a promising future for minimally invasive liver surgery [63].

## Liver transplantation for HCC

### Evolution of liver transplantation and selection criteria

Liver transplantation has been established as a definitive treatment for early-stage hepatocellular carcinoma since the landmark introduction of the Milan criteria in 1996. Under these criteria, which defined eligibility as either a single tumor ≤ 5 cm or up to three tumors ≤ 3 cm without macrovascular invasion, excellent outcomes with 4-year survival rates of 75% have been achieved [64]. The success of the Milan criteria has prompted the exploration of expanded parameters. More recently, the “up-to-seven” criteria (where the sum of the size of the largest tumor [in cm] and the number of tumors does not exceed seven) has shown promising results, achieving 5-year survival rates of 71.2% in carefully selected patients without microvascular invasion [65]. In Asia, where living donor liver transplantation (LDLT) predominates, the “5–5–500 rule” (tumors ≤ 5 cm in size, tumor number ≤ 5, and Alpha-fetoprotein: AFP level ≤ 500 ng/mL) has shown promising results in regional variations in organ availability have led to different transplantation approaches [66, 67]. Western countries primarily utilize deceased donor liver transplantation (DDLT), employing MELD scores with HCC exception points for allocation. However, wait-list dropout due to tumor progression remains a significant challenge, necessitating careful patient selection and bridging therapies. In Asia, where organ scarcity is more pronounced, living donor liver transplantation (LDLT) predominates. While LDLT offers advantages in timing and waiting period, it requires meticulous donor evaluation, including volumetric assessment. The donor operation carries a reported mortality risk of 0.2–0.5%, necessitating careful ethical considerations [68, 69].

### Prevention and management of HCC recurrence after liver transplantation

FDG-PET imaging provides valuable predictive insights for selecting liver transplant candidates in HCC [70]. Specific PET/CT metabolic parameters—such as the tumor-to-background ratio, metabolic tumor volume, and total lesion glycolysis—are independent predictors of microvascular invasion and post-transplant recurrence, with higher values linked to significantly poorer recurrence-free survival [71, 72]. Liquid biopsy, through the analysis of circulating tumor cells (CTCs), circulating tumor DNA (ctDNA), and other genomic biomarkers in blood, offers a non-invasive method to evaluate tumor biology [72]. Detection of specific CTC subtypes, particularly those with mesenchymal markers like Vimentin [73], is associated with elevated recurrence risk and poorer prognosis. Integrating liquid biopsy markers with conventional criteria could thus improve early recurrence detection and enhance the precision of candidate selection for liver transplantation in HCC.

Immune checkpoint inhibitors (ICIs) have revolutionized the treatment of advanced hepatocellular carcinoma (HCC), but their application in liver transplantation settings requires careful consideration of timing, patient selection, and potential risks, presenting unique challenges. In the pre-liver transplantation setting, ICIs are being investigated as downstaging and bridging therapies. Tabrizian et al. reported mild acute rejection in one out of nine patients treated with nivolumab before liver transplantation [74]. However, a multi-center study from China found a [75], with an interval shorter than 30 days between the last ICI dose and liver transplantation, identified as a predictor of rejection. Interim results of ongoing trials investigating the safety and efficacy of ICIs in the pre-LT setting have shown outcomes without acute allograft rejection and improved recurrence-free survival. However, the timing of ICI cessation before transplantation is crucial, with a recommended washout period of at least 4–6 weeks. In the post-liver transplantation setting, ICIs are associated with a significant risk of graft loss. A systematic review found a 28.8% rejection rate among patients treated with ICIs for de novo malignancies after liver transtation [76]. Currently, tyrosine kinase inhibitors are recommended for advanced HCC recurrences after liver transplantation, while ICIs may be considered a salvage option.

## Impact of preoperative and postoperative therapies on surgical outcomes

### Preoperative therapy: enhancing surgical eligibility and outcomes

Preoperative therapies, particularly downstaging chemotherapy, are pivotal in increasing the eligibility of hepatocellular carcinoma (HCC) patients for surgical intervention [77, 78]. Conversion therapy, also known as downstaging, aims to reduce the tumor burden in initially unresectable HCC cases to make surgery feasible. Recent studies have demonstrated promising results using various systemic therapies. The LENS-HCC trial [79], a multicenter phase 2 study, evaluated lenvatinib, an oral multikinase inhibitor targeting VEGF receptors 1–3 and other pathways, as preoperative therapy for advanced HCC. Among 49 patients with factors suggesting poor prognosis (such as macroscopic vascular invasion, extrahepatic metastasis, or multinodular tumors), the trial achieved a remarkable surgical resection rate of 67.3% after 8 weeks of lenvatinib therapy, with a particularly high rate of 76.2% in oncologically unresectable cases. The therapy demonstrated both safety and efficacy, with an objective response rate of 37.5% based on mRECIST criteria and a one-year survival rate of 75.9%. Other approaches, such as immune checkpoint inhibitors (ICIs) combined with vascular endothelial growth factor (VEGF) inhibitors, have also shown promising response rates in tumor downsizing. For instance, a trial evaluating atezolizumab and bevacizumab reported an objective response rate of 27%, with some patients achieving sufficient reduction in tumor size to qualify for surgical resection [80]. This strategy holds the potential to extend curative surgery to a broader patient cohort, although further evidence from larger trials is needed to establish definitive efficacy.

### Adjuvant therapy: reducing postoperative recurrence rates

Postoperative recurrence remains a significant obstacle in HCC, with up to 70% of patients experiencing relapse within five years of surgery.

Despite numerous phase III trials, adjuvant therapies like retinoids, vitamin K2 [81], interferon-alpha [82], and sorafenib [83] have generally failed to show significant benefits in reducing HCC recurrence post-resection or ablation, with some positive findings lacking validation in other populations. Recent studies have shown improved recurrence-free survival observed in patients receiving hepatic intra-arterial chemotherapy with FOLFOX, adjuvant TACE, and adoptive cell therapy with cytokine-induced killer cells [84] using peripheral blood mononuclear cells with IL-2 and an anti-CD3 antibody [85]. However, these findings require careful validation.

Antiviral therapies have further demonstrated benefits in reducing recurrence rates, especially in hepatitis B and C-related HCC.

The efficacy of adjuvant atezolizumab combined with bevacizumab in high-risk HCC patients initially showed promise, with early analysis demonstrating improved recurrence-free survival compared to active surveillance (HR 0.72; 95% CI: 0.56–0.93; p = 0.012) [86]. However, updated data from 2024 revealed that this initial benefit was not maintained in longer follow-up (HR 0.90; 95% CI: 0.72–1.12), suggesting that this combination may not be an optimal adjuvant strategy for high-risk HCC patients [87].

### Challenges and future directions in conversion and adjuvant therapies

While downstaging chemotherapy and adjuvant therapies present promising avenues for improving HCC outcomes, several challenges remain. Effective biomarkers are essential for identifying candidates who benefit most from conversion or adjuvant treatments. Additionally, managing adverse effects, especially in patients with underlying liver disease, is critical to the safe administration of these therapies. Future research holds promise for advancing precision medicine through multidisciplinary approaches that integrate various systemic and locoregional therapies to better tailor treatments to the individual patient needs.
